# Genetic Variation for Economically Important Traits in *Cupressus lusitanica* in New Zealand

**DOI:** 10.3389/fpls.2021.651729

**Published:** 2021-06-08

**Authors:** Ahmed Ismael, Jaroslav Klápště, Grahame T. Stovold, Kane Fleet, Heidi Dungey

**Affiliations:** Scion, Rotorua, New Zealand

**Keywords:** *Cupressus lusitanica*, heritability, genetic correction, canker, disease resistance

## Abstract

Increasing productivity and tolerance against cypress canker disease is an important goal in the Mexican white cypress breeding program in New Zealand, and screening has been in place since 1983. Cypress canker disease is caused by *Seiridium cardinale* and *Seiridium cupressi*, the current study presents the results of two progeny trials within the breeding program in the North Island of New Zealand. The trials were established as open-pollinated progeny tested and were assessed for diameter at breast height, branch size, canker severity score, malformation score, and stem straightness score and acceptability score. Heritability estimates were moderate ranging from 0.21 to 0.41 for diameter at breast height and from 0.14 to 0.31 for canker severity score. Stem form attributes showed heritability from 0.08 (malformation) to 0.38 (straightness). No trait showed any significant G × E interaction between investigated sites. This was supported by the very strong genetic correlations estimated between the traits recorded in Welcome Bay and Matata trials. Unfavourable genetic correlations ranging from 0.25 to 0.46 were found between diameter at breast height and canker severity score, indicating that the continued selection for genotypes with improved diameter at breast height would also increase susceptibility to cypress canker. Additionally, unfavourable genetic correlations ranging from 0.52 to 0.73 were found between branch size and diameter at breast height and should be considered in selection programs. The moderate heritability estimated for canker severity score indicates that breeding values for this trait could be predicted with acceptable accuracy and included in the breeding program for *Cupressus lusitanica*, enabling the identification of genotypes with tolerance to canker severity to be deployed to locations where cypress canker is present in New Zealand.

## Introduction

The overall objective of genetic improvement in forestry breeding programs is to increase the productivity and economic value of planted forests. Generally, such programs aim to develop economically efficient genetically improved tree genotypes by maximising genetic gain per time unit at the cheapest cost (White et al., 2007). The cypresses are evergreen coniferous trees belonging to the family *Cupressaceae*. In New Zealand, cypresses provide an excellent opportunity for tree improvement to maximise selection and production of durable, scented timber. Cypresses have been planted in New Zealand for over 150 years, and account for an estimated area of 10,000 ha (Bulman and Hood, 2018). Two cypress species have been prioritised for New Zealand: Mexican white cypress (*Cupressaceae lusitanica*) and Monterey cypress (*Cupressus macrocarpa* Hartw.). This is because their timber is well characterised for its durability, low shrinkage rate, even density gradient, drying stability and overall appearance (Miller and Knowles, 1996; Nicholas, 2006). However, *C. lusitanica* is increasing in importance because of its preference over *C. macrocarpa* on warm sites due to its lower susceptibility to cypress canker disease (Van Der Werff, 1988). *C. lusitanica* is a species of cypress found naturally in Central North America, Mexico, Guatemala, and Honduras (Miller et al., 1990).

In recent years, the global spread of non-native pathogens has continued to increase rapidly due to trade and the movements of people (Sikes et al., 2018). This could have a major effect on the health and productivity of native forests and forest plantations, potentially causing significant ecological and economic damage (Lovett et al., 2016; Sniezko and Koch, 2017). The introduction of exotic tree species in the absence of pathogen-host coevolution (Woolhouse et al., 2002; Occhipinti, 2013) can result in devastating biological impacts on the host species (Gonthier and Garbelotto, 2013; Santini et al., 2013). Furthermore, economic damage due to loss of productivity and cost of disease control can amount to millions or billions of dollars per year (van der Pas, 1981; van der Pas et al., 1984; Pimentel et al., 2005; Watt et al., 2012). Fungal disease is one of the major risks impacting the profitability of forestry plantations, mainly because dense plantations are ideal for infection by fungal pathogens (Burdon, 2008).

Cypress canker is a pandemic disease which has caused severe mortality worldwide in many species and hybrids of *Cupressaceae* (Graniti, 1998; Danti et al., 2013a). The disease is the most economically important and major limiting factor for the production of cypresses as a commercial crop in New Zealand. Damage is caused by the infection of stems and branches by two fungi, *Seiridium cupressi* (previously misidentified in New Zealand as *Seiridium unicorne*) (Cooke and Ellis) Sutton and *Seiridium cardinale* (Wagener) Sutton and Gibson (Van Der Werff, 1988; Nicholas, 2006; Danti and Della Rocca, 2017). Cypress cankers have been found throughout New Zealand since 1930 (Nicholas, 2006), and there are no clear effects of host age class or climate on disease spread due to the large variation in disease incidence and severity (Bulman and Hood, 2018). However, there was a tendency for the disease severity to be higher in warmer regions and older stands in forest plantations, making it more widespread in Northland and Auckland (North of New Zealand’s North Island) (Van Der Werff, 1988; Nicholas, 2006; Bulman and Hood, 2018). *S. cardinale* population in New Zealand includes two distinct genetic groups. An older group was identified in California that likely to be native and could be originally introduced from California before 1933 (Della Rocca et al., 2019). While a more recent group was identified in the Mediterranean region and could be introduced from Europe before 1982 (Della Rocca et al., 2019). It has been reported that the Californian groups are reproducing clonally and sexually, while the Mediterranean groups are only reproducing clonally (Della Rocca et al., 2013). The economic impact of cypress canker on New Zealand forests is significant, and cypresses have been avoided for planting because of the threat of malformation and death by cypress canker (Van Der Werff, 1988). The disease is characterised by reddish-brown necrotic spots in the early stages of infection, often associated with resin emission (Van Der Werff, 1988). The necrosis spreads through the bark and cracks appear, from which resin flows (Danti and Della Rocca, 2017). As a result, in young trees, the canker can destroy the stem or branches rapidly. In contrast, in adult trees, the infected branches eventually die due to the damage caused by the cankers and from a toxin produced by the fungi (Van Der Werff, 1988; Danti et al., 2013a; Danti and Della Rocca, 2017). Another important diagnostic feature is the appearance of the asexual fruiting bodies of the fungus that develop on the surface of the necrotic bark during disease development (Van Der Werff, 1988; Danti and Della Rocca, 2017). The asexual spores are produced in small, black fruiting bodies which erupt through the bark surface when weather conditions are moist and warm. Spores are dispersed through the stem and branches, or from tree to tree through water splash or wind, or by insects and birds (Van Der Werff, 1988; Danti et al., 2013a; Danti and Della Rocca, 2017). Control methods such as spraying of fungicides are not practically feasible in forest plantations. Alternative method for controlling cypress canker is to select and screen for cypress clones resistant to the disease.

Breeding for genotypes with tolerance to cypress canker, growth and form traits has been underway since 1983. Impact of cypress canker has been found to be under moderate genetic control, indicating that targeted selection might improve this trait. For example, the narrow-sense heritability for canker severity ranged from 0.27 to 0.30 using seedling or clonally replicated progenies in two trials of 6-years-old *C. lusitanica* in New Zealand (Dungey et al., 2013). Furthermore, Gea and Low (1997) reported narrow-sense heritability estimates ranging from 0.04 to 0.20 for canker severity in two trials of 7- and 10-years-old *C. macrocarpa* in New Zealand.

The main objectives of the breeding programme for the primary cypress species in New Zealand, *C. macrocarpa* and *C. lusitanica*, include selection for growth, form, branching, and tolerance to canker severity. The best genotype based on these traits are selected in every tested family, and seeds collected from these selections are used to create the next generation of the breeding population (Van Der Werff, 1988; Miller and Knowles, 1996; Dungey et al., 2015).

Currently, there is little documentation of genetic relationships between canker severity, growth and form traits, age-age correlations and genotype by environment interactions (G × E) in *C. lusitanica* breeding program. For example, Dungey et al. (2013) reported high genetic correlations (0.73) between DBH and branch size, and low between DBH and canker severity (−0.08), and between canker severity and branch size (−0.07) in 6 years-old *C. lusitanica* trial in New Zealand. The same authors found no evidence of significant G × E interaction for DBH between Paengaroa and Katikati forests in New Zealand.

In the current study, canker severity, growth, and form traits were assessed in two progeny trials established within the *C. lusitanica* breeding program in New Zealand. Our objectives were: (1) to obtain information about the genetic complexity of these traits through the estimation of variance components and heritability; (2) to investigate the associations between economically important traits and tolerance to canker severity through genetic correlation analysis; and (3) to investigate persistence in productivity and tolerance to canker severity across different environments, through genotype by environment interaction (G × E) analysis using progeny trials located on the North Island of New Zealand.

## Materials and Methods

### Description of Trials

Two progeny trials within the *C. lusitanica* breeding population were used to estimate the genetic parameters for traits across two sites located in the North Island of New Zealand (Table 1). Both trials were randomised incomplete block designs with an “optimal design” (Butler, 2013) as a single-tree plot. The Welcome Bay trial contained 103 open-pollinated families with 3,360 trees (30 trees per family) in total. This trial was assessed for diameter at breast height (DBH), branch size, canker severity score, malformation score, stem straightness and acceptability score. The Matata trial consisted of 110 open-pollinated families and with 3,600 trees (30 trees per family) in total. This trial was assessed for DBH, canker severity score and acceptability score. Before the assessment for canker severity score, a field expert (i.e., pathologist/assessor) visited the two sites to confirm the existence of cypress canker by visual symptoms. Welcome Bay is at low altitude (250 m above sea level), sloping at about 20°–25°. By contrast, Matata is south facing and at higher altitude (300 m above sea level). Description of the traits assessed in the two trials are summarised in Table 1.

**TABLE 1:** Description of progeny trials at Welcome Bay and Matata in New Zealand.

Trial	Welcome Bay	Matata
Establishment date	July, 2007	July, 2007
Latitude	37°44′20.122′′	37°59′33.548′′
Longitude	176°16′5.58′′	176°39′44.64′′
Area (ha)	3.00	3.20
Altitude (m)	50	347
No. planted trees	3,360	3,600
No. families	103	110
No. replications	30	30
No. blocks	120	120
No. trees per block	28	28
Tree spacing (m)	3 × 3	3 × 3
Design	Incomplete block design with single-tree plots	

The studied populations represent the third generation of the breeding program. There was a total of 100 parents shared between both sites.

The New Zealand *C. lusitanica* breeding population was initiated in 1982 with 80 selections of well-formed and vigorous trees in New Zealand stands, 25 other selections received from selected trees in Kenya and Colombia, and three seedlots of mixed parentage, resulting in a total of 110 selections.

### Definition of Traits Assessed at the Welcome Bay

(1) Diameter at breast height at 60 months of age (DBH60), measured in millimetres.
(2) Branch size at 60 months of age (BRS60), was scored from 1 to 5: as the average branch size across the crown, discounting malformations, where 1 = 1 cm and 5 = 5 cm (smaller is better).
(3) Canker severity score at 60 months of age (CNK60), was scored from 0 to 3 (0 = no canker symptoms, 1 = hollows on the stem that may have been caused by canker, 2 = hollows with evident dead cambium, 3 = large patch of dead cambium).
(4) Malformation score at 60 months of age (MAL60), was scored from 1 to 9 (1 = multiple forking, 9 = perfect).
(5) Stem straightness score at 60 months of age (STR60), was scored from 1 to 8 (1 = bad, 8 = excellent).
(6) Acceptability score at 118 months of age (AC2118) as combination between growth and form and scored from 0 to 2, (0 = unacceptable, 1 = acceptable, 2 = candidate plus tree).
(7) Canker severity score at 118 months of age (CNK118), scored as above.
(8) Diameter at breast height at 118 months of age (DBH118), measured as above.

### Definition of Traits Assessed at Matata

(1) Canker severity score at 119 months of age (CNK119), scored as above.
(2) Diameter at breast height at 119 months of age (DBH119), measured as above.
(3) Acceptability score at 119 months of age (AC2119), scored as above.

## Statistical Analysis

### Estimation of Variance Components and Genetic Parameters

Genetic analyses were performed with the average information restricted maximum likelihood (AI-REML) algorithm implemented in the ASReml-R v.3 statistical package (Butler et al., 2009). The single-trait analysis was performed to estimate variance components and narrow-sense heritability for each trait separately, whereas multi-trait analysis was performed to estimate genetic correlations between the traits as well as between sites.

All traits were analyzed using the following individual tree linear mixed model (Borralho, 1995):

(1) $$Y=X\,\beta +Z_{r}+Z_{b}+{\text{Z}}_{p}+Z\,a+e$$

Where ***Y*** is the matrix of phenotypes analysed; β is the vector of fixed effects containing the overall mean; ***r*** is the vector of random replication effects following $\text{var}\,\left(r\right)∼\text{N}\,\left(0,I\,\sigma_{r}^{2}\right)$, where $\sigma_{r}^{2}$ is replication variance and ***I*** is identity matrix; ***b*** is the vector of random block effects following $\text{var}\,\left(b\right)∼\text{N}\,\left(0,I\,\sigma_{b}^{2}\right)$, where $\sigma_{b}^{2}$ is block variance; ***p*** is the vector of random provenance effects following $v\,a\,r\,\left(p\right)∼\text{N}\,\left(0,I\,\sigma_{p}^{2}\right)$ where $\sigma_{p}^{2}$ is provenance variance; *a*_*l*_ is the vector of ploygenic random additive genetic effect which was assumed to be normally distributed following $\text{var}\,\left(a\right)∼\text{N}\,\left(0,A\,\sigma_{a}^{2}\right)$, where $\sigma_{a}^{2}$ is the additive genetic variance, and ***A*** is the pedigree-based average numerator relationship matrix (Wright, 1922), and *e* is the vector of random residual effects.

Preliminary analyses were performed to test the significance of the improvement achieved through using a spatial autoregressive mixed model over a traditional mixed model without the autoregressive term (i.e., non-spatial), using a likelihood ratio test (LRT). The log-likelihoods of the two models were compared against the chi-square distribution (Dutkowski et al., 2006). Spatial models included the first-order autoregressive random error terms on the rows (row) and columns (col) directions. The residual structure (*R*) was divided into spatially dependent (ξ) and independent (η) error terms as $R=\sigma_{\xi }^{2}\,\left[\mathrm{AR1}\,\left(\rho_{c\,o\,l}\right)⊗\mathrm{AR1}\,\left(\rho_{r\,o\,w}\right)\right]+I\,\sigma_{\eta }^{2}$, where $\sigma_{\xi }^{2}$ is the spatial variance, ⊗ is the Kronecker product and AR1(*ρ*) represents a first-order autoregressive correlation matrix for rows and columns where *ρ* is the autocorrelation parameter and $\sigma_{\eta }^{2}$ is independent residual variance. For the traits recorded at age 118 months in Welcome Bay, the spatial models did not converge successfully due to the insufficient number of observations, and we only reported results from a linear mixed model without the autoregressive term.

Variance component estimates from the single-trait analysis were used to estimate the narrow-sense heritability (*h*^2^) as

(2) $$h^{2}=\frac{\sigma_{a}^{2}}{\sigma_{a}^{2}+\sigma_{\eta }^{2}}$$

Where $\sigma_{a}^{2}$ is the additive genetic variance, and the independent error term σ_η_^2^.

Genotype by environment interaction (G × E) among traits (DBH, CNK, and AC2) assessed in Welcome Bay and Matata forest was measured as the departure from 1 of the genetic correlation between same traits across different environments (sites). Model 1 was therefore extended to a bivariate model to estimate the genetic correlation between Welcome Bay and Matata, with values below 0.8 indicating the presence of a significant G × E interaction (Robertson, 1959).

Within each site, the model (1) was extended to a bivariate analysis to estimate the pairwise genetic correlations between traits. The variance-covariance structure for this model is

(3) $$\begin{array}{c} \begin{array}{c} \left[\begin{array}{c} R_{1}\\ R_{2} \end{array}\right] \end{array}∼N\,\left[\begin{array}{c} \begin{array}{cc} I\,\sigma_{R_{1}}^{2} & 0\\ 0 & I\,\sigma_{R_{2}}^{2} \end{array} \end{array}\right],\begin{array}{c} \left[\begin{array}{c} B_{1}\\ B_{2} \end{array}\right] \end{array}∼N\,\left[\begin{array}{c} \begin{array}{cc} I\,\sigma_{B_{1}}^{2} & 0\\ 0 & I\,\sigma_{B_{2}}^{2} \end{array} \end{array}\right],\begin{array}{c} \left[\begin{array}{c} P_{1}\\ P_{2} \end{array}\right]∼N\,\left[\begin{array}{c} \begin{array}{cc} I\,\sigma_{P_{1}}^{2} & 0\\ 0 & I\,\sigma_{P_{2}}^{2} \end{array} \end{array}\right], \end{array}\\ \begin{array}{c} \left[\begin{array}{c} a_{1}\\ a_{2} \end{array}\right] \end{array}∼N\,\left[\begin{array}{c} \begin{array}{cc} A\,\sigma_{a_{1}}^{2} & A\,\sigma_{a_{1}\,a_{2}}\\ A\,\sigma_{a_{2}\,a_{1}} & A\,\sigma_{a_{2}}^{2} \end{array} \end{array}\right],\begin{array}{c} \left[\begin{array}{c} e_{1}\\ e_{2} \end{array}\right] \end{array}∼N\,\left[\begin{array}{c} \begin{array}{cc} I\,\sigma_{e_{1}}^{2} & I\,\sigma_{e\,1\,e\,2}\\ I\,\sigma_{e\,2\,e\,1} & I\,\sigma_{e_{2}}^{2} \end{array} \end{array}\right] \end{array}$$

Where all the design terms were defined as the model (1), σ_*a_1 a_2*_ is the additive genetic covariance between the 1st and 2nd trait and σ_*e_1 e_2*_ is the residual covariance between the 1st and 2nd trait.

Genetic correlations (*r*_*g_12*_) between traits were estimated as:

(4) $$r_{g_{12}}=\frac{\sigma_{a\,1\,a\,2}}{\sqrt{\sigma_{a\,1}^{2}}\,\sigma_{a\,2}^{2}}$$

Significance levels of genetic correlations were assessed as deviations of >1.645 × standard error from zero, where the value 1.645 corresponds to the one-sided 5% cut off point of the normal distribution (*P* < *0.05*).

Approximate standard errors of estimates of the heritability for each trait and genetic correlations between traits were calculated from the standard errors of the covariance components using a Taylor series expansion approximation, implemented in the ASReml-R package (Butler et al., 2009).

## Results

### Descriptive Statistics

The number of trees assessed per trait, overall means for each trait, as well as minima, maxima, individual-tree standard deviations and percentage of infected trees, have been summarised in Tables 2, 3. In general, trees showed greater growth at Welcome Bay, as DBH118 had a higher mean value compared with trees at Matata. Also, the mean value of the acceptability score for trees at Welcome Bay was higher than at Matata. The severity of cypress canker infection (mean disease score) was the highest at Matata (mean score = 0.74) at 119 months of age (Table 3), and the lowest was at 60 months of age at Welcome Bay (mean score = 0.46) (Table 2). The severity of cypress canker at Welcome Bay at 118 months of age was similar to the severity of cypress canker at Matata at 119 months of age (mean score = 0.71). The disease incidence expressed as the percentage of trees infected by disease (disease score > 0) was highest at Matata (46% infected trees). In contrast, the lowest disease incidence was recorded at Welcome Bay at 60 months of age (24% infected trees).

**TABLE 2:** Number of observations, mean, standard deviation, minimum and maximum of all traits recorded at Welcome Bay.

Trait	*N*	Mean	SD	Min	Max
BRS60	2,753	2.73	0.82	1	5
CNK60	2,753	0.46	0.91	0	3
DBH60	2,753	128.3	27.9	45	218
MAL60	2,749	7.83	2.38	1	9
STR60	2,753	5.79	1.41	1	8
AC2118	1,876	0.58	0.56	0	2
CNK118	1,906	0.71	1.02	0	3
DBH118	1,884	214.5	42.7	19	345
					

**TABLE 3:** Number of observations, mean, standard deviation, minimum and maximum of all traits recorded at Matata sites.

Trait	*N*	Mean	SD	Min	Max
AC2119	2,664	0.54	0.52	0	2
CNK119	2,666	0.74	0.96	0	3
DBH119	2,665	182.8	36.8	63	330

### Variance Components and Heritability Estimates

**FIGURE 1:**
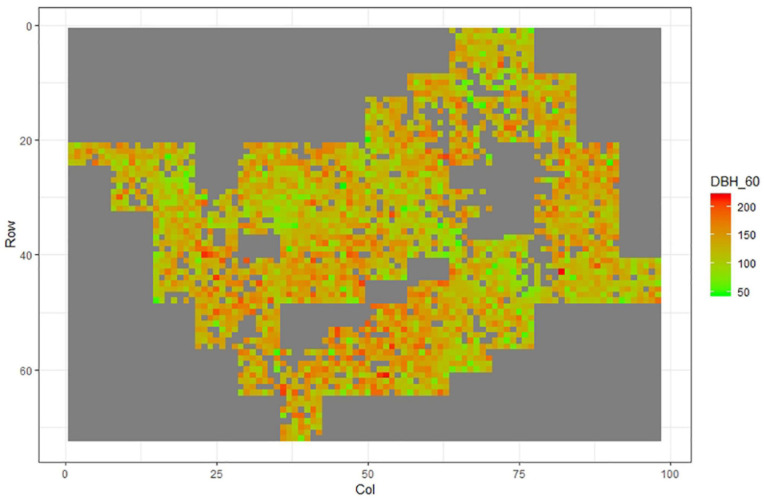
Spatial distribution of DBH60 measured at Welcome Bay site.

**FIGURE 2:**
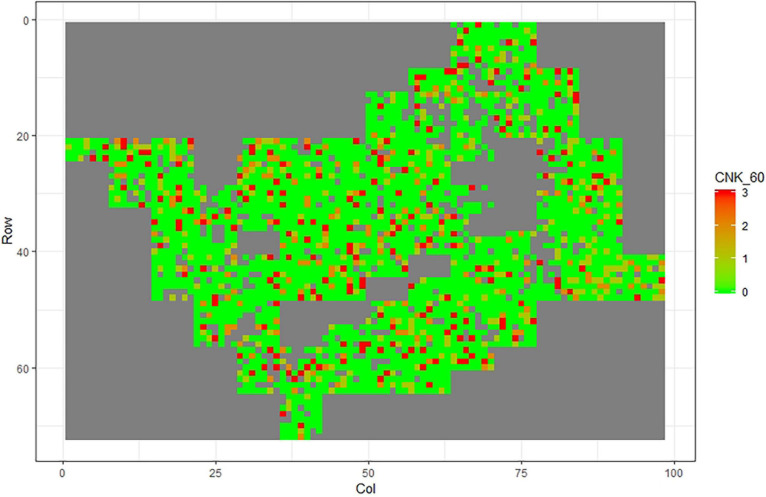
Spatial distribution of CNC60 measured at Welcome Bay site.

**FIGURE 3:**
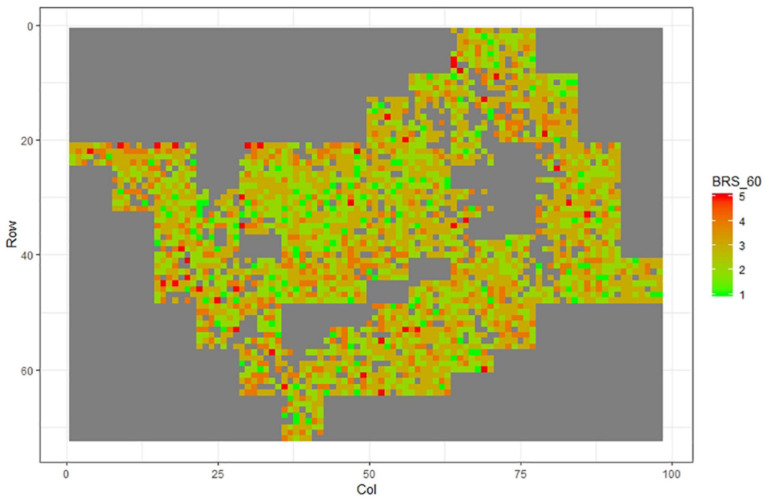
Spatial distribution of BRS60 measured at Welcome Bay site.

**FIGURE 4:**
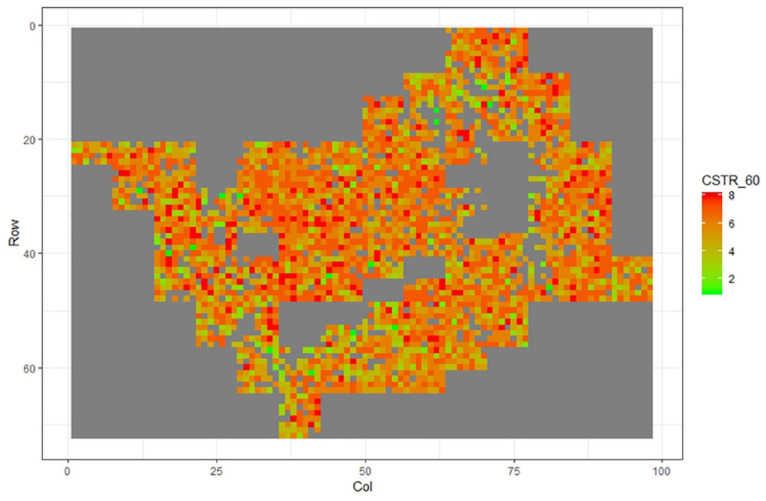
Spatial distribution of CSTR60 measured at Welcome Bay site.

**FIGURE 5:**
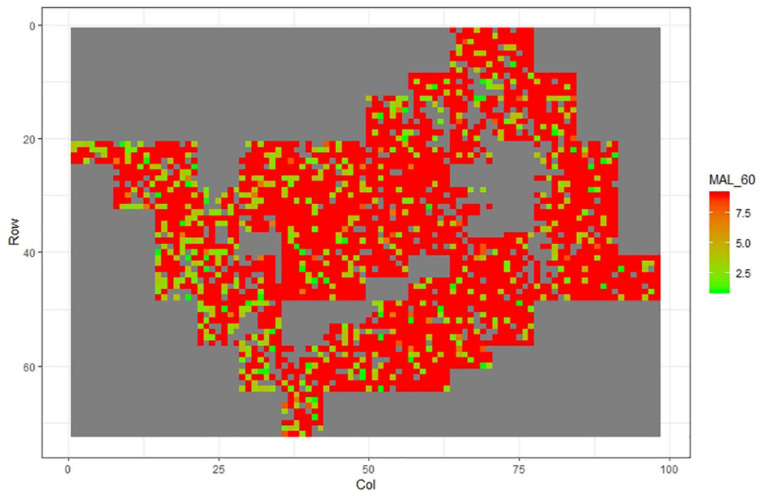
Spatial distribution of Mal60 measured at Welcome Bay site.

**FIGURE 6:**
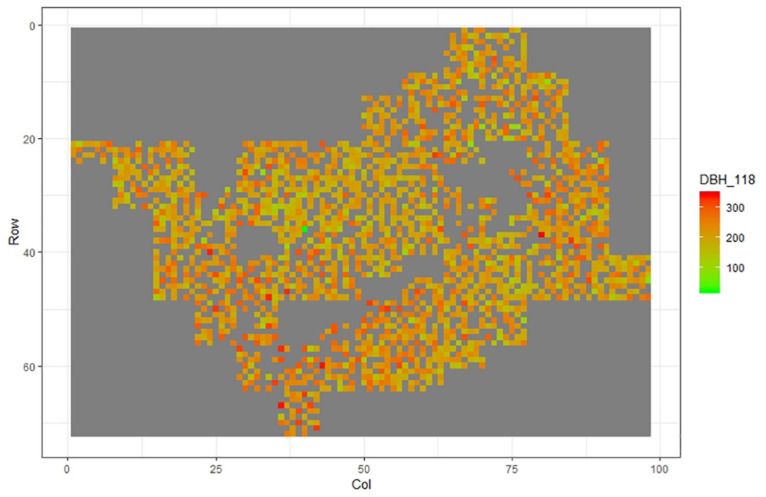
Spatial distribution of DBH118 measured at Welcome Bay site.

**FIGURE 7:**
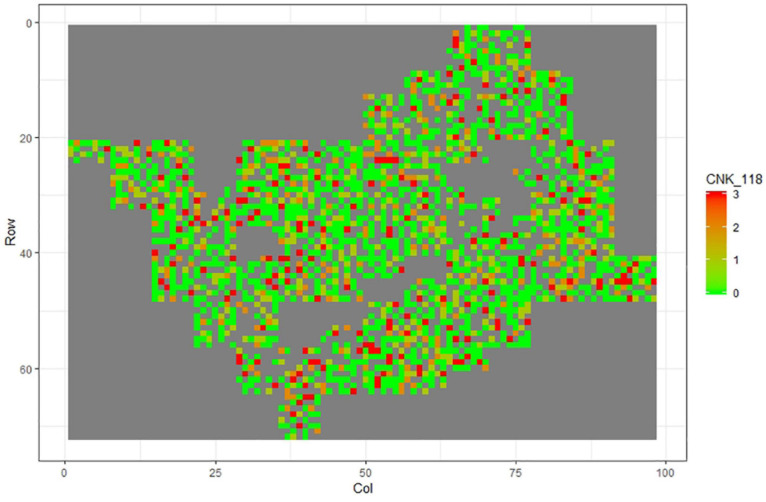
Spatial distribution of CNK118 measured at Welcome Bay site.

**FIGURE 8:**
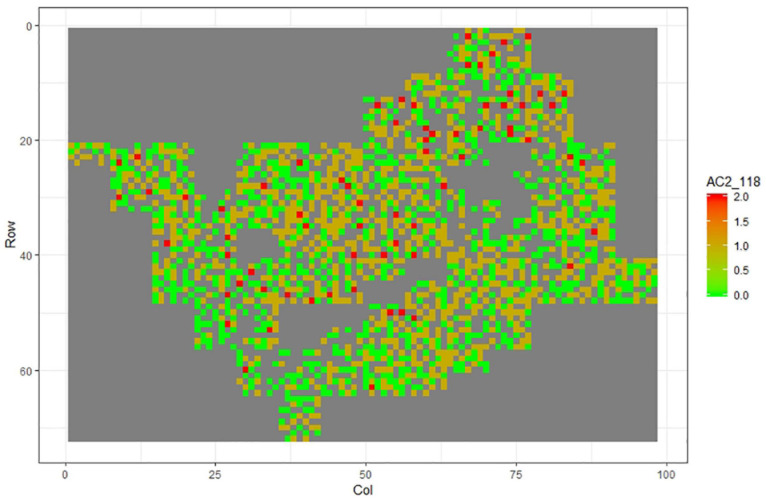
Spatial distribution of AC2118 measured at Welcome Bay site.

**FIGURE 9:**
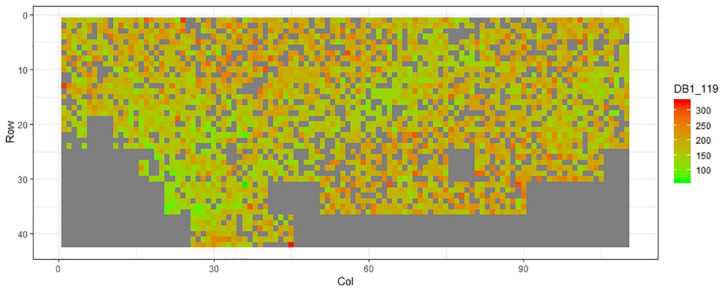
Spatial distribution of DBH119 measured at Matata site.

**FIGURE 10:**
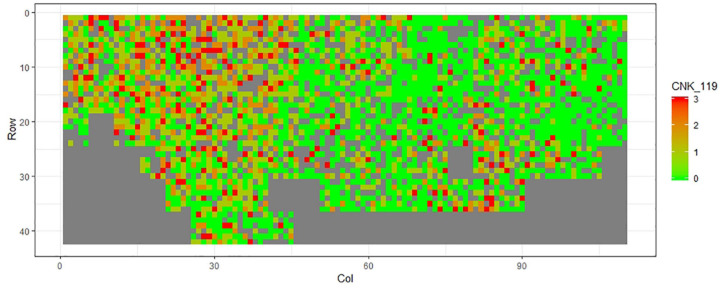
Spatial distribution of CNK119 measured at Matata site.

**FIGURE 11:**
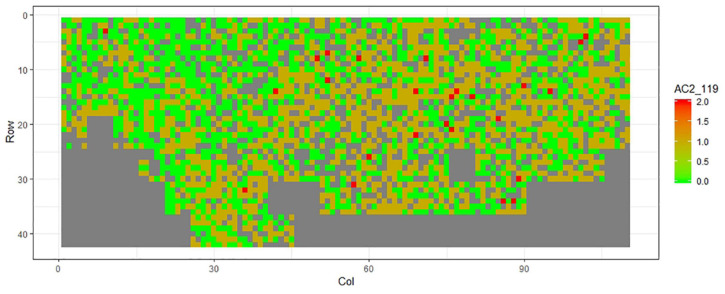
Spatial distribution of AC2119 measured at Matata site.

Figures 1–11 show the spatial distribution of the phenotypic expression for each trait in each site of the experiment, with each square representing an individual tree within the row/column design of each trial. Canker severity score at Matata showed the highest spatial residual variation (Figure 10), with more discernible clumps of infection compared to those at Welcome Bay. Canker severity in Welcome Bay showed patches of infection mainly in the middle of the trial and fewer patches toward the edges in Figures 2, 7. In contrast, a big patch of infection appeared in the top left of the Matata (Figure 10). The preliminary analyses showed that the spatial autoregressive mixed model performed were significantly better than the non-spatial mixed models based on the log likelihood comparisons (chi-square test).

An overview of variance components and narrow-sense heritability estimates for each of the traits at each trial is shown in Tables 4, 5. The provenance was associated with significant variation of DBH (i.e., 20–35% of the total variation), and STR (i.e., 37% of the total variance). All narrow-sense heritability estimates for DBH were moderate in both sites and varied from 0.21 at Matata to 0.41 at Welcome Bay. Similarly, branching size at Welcome Bay had moderate heritability (0.32), whereas heritability estimates for canker severity were roughly twice as high at Welcome Bay (0.31) as estimates from Matata (0.14). On the other hand, heritability estimates for acceptability score tended to be weak and did not differ substantially among sites (Tables 4, 5). However, the heritability estimate for the Matata (0.17) was slightly higher than the estimate from Welcome Bay (0.14). Stem straightness was moderately heritable (*h*^2^ = 0.38), whereas the lowest heritability estimate was for the malformation score (0.08).

**TABLE 4:** Estimates of variance components and narrow-sense heritability (*h*^2^) with approximate standard errors in parentheses for all traits measured at Welcome Bay site.

Trait	$\sigma_{R}^{2}$	$\sigma_{B}^{2}$	$\sigma_{P}^{2}$	$\sigma_{a}^{2}$	$\sigma_{\xi }^{2}$	$\sigma_{\eta }^{2}$	*h*^2^
BRS60	0	0	0.01	0.20	0.05	0.42	0.32 (0.06)
DBH60	0.68	5.94	103.73	253.08	136.99	365.20	0.41 (0.08)
CNK60	0	0.004	0	0.25	0.005	0.58	0.30 (0.06)
MAL60	0.19	0.05	0	0.43	NA	5.01	0.08 (0.03)
STR60	0.05	0	0.02	0.65	0.23	1.06	0.38 (0.07)
AC2118	0.01	0	0	0.04	NA	0.27	0.14 (0.05)
CNK118	0	0	0	0.33	NA	0.72	0.31 (0.07)
DBH118	109.81	42.71	293.4	621.13	NA	1036.4	0.37 (0.08)

*$\sigma_{R}^{2}$, replications variance; $\sigma_{B}^{2}$, block variance; $\sigma_{P}^{2}$, provenance variance; $\sigma_{a}^{2}$, additive genetic variance; $\sigma_{\xi }^{2}$, spatially dependent error variance; $\sigma_{\eta }^{2}$, independent error variance; *h*^2^, narrow-sense heritability; NA, the spatial analysis didn’t converge during the analysis.*

**TABLE 5:** Estimates of variance components and narrow-sense heritability (*h*^2^) with approximate standard errors in parentheses for all traits measured at Matata site.

Trait	$\sigma_{R}^{2}$	$\sigma_{B}^{2}$	$\sigma_{P}^{2}$	$\sigma_{a}^{2}$	$\sigma_{\xi }^{2}$	$\sigma_{\eta }^{2}$	*h*^2^
CNK119	0	0	0	0.10	0.29	0.63	0.14 (0.04)
DBH119	3.51	0.37	97.75	218.25	263.45	809.73	0.21 (0.05)
AC2119	0	0	0	0.04	0.02	0.20	0.17 (0.05)

*$\sigma_{R}^{2}$, replications variance; $\sigma_{B}^{2}$, block variance; $\sigma_{P}^{2}$, provenance variance; $\sigma_{a}^{2}$, additive genetic variance; $\sigma_{\xi }^{2}$, spatially dependent error variance; $\sigma_{\eta }^{2}$, independent error variance; *h*^2^, narrow-sense heritability; NA, the spatial analysis didn’t converge during the analysis.*

### Genetic Correlations Between Traits

**FIGURE 12:**
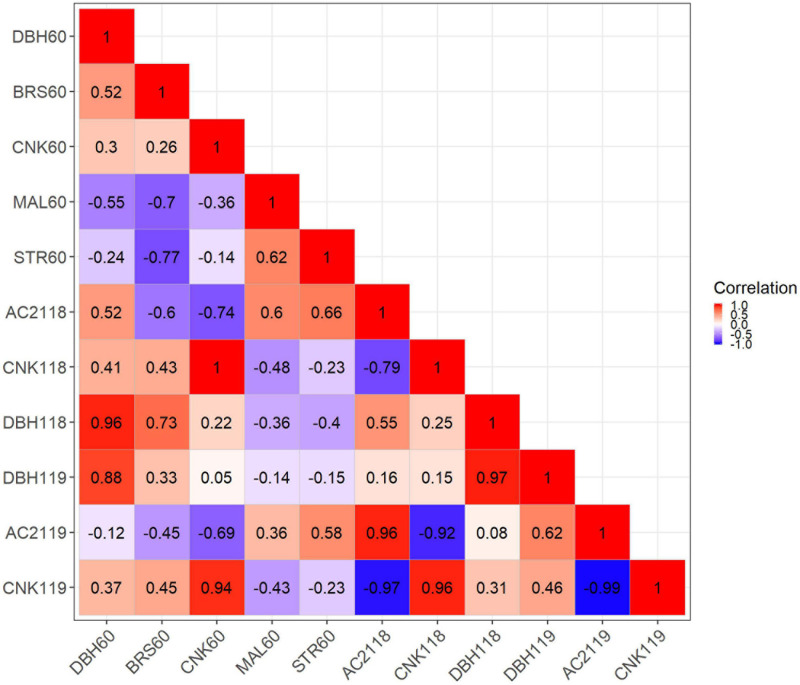
Genetic correlation for the traits measured Welcome Bay and Matata site.

The additive genetic correlations between traits recorded at Welcome Bay and Matata were very strong and close to one (>0.85), indicating little evidence of G × E for diameter, acceptability score, and canker severity score (Figure 12). The additive genetic correlations between DBH, branch size, and acceptability score were moderate to strong (0.52–0.73), indicating that selection for increased DBH will also increase branch size, and trees with larger diameters will have higher acceptability scores. By contrast, the genetic correlation between branch size and acceptability score was negative (−0.60), indicating that trees with larger branch sizes will also have lower acceptability scores (Figure 12).

Additive genetic correlations between growth traits (DBH and branch size) and stem form attributes (malformation score and stem straightness) were moderate but negative (−0.24 to −0.77), indicating that the larger the diameter or branch size, the lower the straightness or malformation score. In contrast, genetic correlations between canker severity score and growth traits were low to moderate (0.22–0.46), indicating that trees with larger DBH and branch size will also be more likely to have more severe canker infection (Figure 12).

Genetic correlations between canker severity score and stem form traits (stem straightness, malformation, and acceptability score) were moderately to strongly negative (−0.23 to −0.99), indicating that trees with high sensitivity to canker will also have a low score for stem straightness, malformation and acceptability. The genetic correlations among form traits were moderate (0.60–0.66), indicating that trees with high scores for stem straightness will also have high scores for malformation (Figure 12).

## Discussion

We assessed the genetic control of canker severity score, growth and form traits in two *C. lusitanica* trials at different ages. All traits were at least weakly to moderately heritable. There was an unfavorable genetic correlation between canker severity scores and growth traits, which needs to be taken into consideration in the cypress breeding program. Canker incidence and severity of expression would be expected to vary between age of trees, year of assessment, and sites. This could be due to variation of climatic variables, sun exposure and microsite characteristics, as well as changing physiology of the trees with age. For example, the severity of canker in the current study was higher than that reported by Dungey et al. (2013). They reported mean canker severity of 0.31 in a 6-years-old *C. lusitanica* progeny trial assessed at closely located sites in the North Island of New Zealand.

### Spatial Variation

Spatial heterogeneity accounted for a significant percentage of the total residual variance of both trials (i.e., 7–28% of the total variances, depending on the trait). This could be due to microsite effects, particularly in the distribution of humidity and nutrients within plantation (Li et al., 2003; Li and Yang, 2004). Thus, accounting for the spatial component of the residual variance is crucial as it can lead to substantial improvements of genetic parameter estimates (Dutkowski et al., 2002, 2006).

Genetic correlations between diameter, acceptability, and canker severity score recorded at Welcome Bay and Matata were strong (0.88–01), indicating weak G × E interactions, with relatively little G × E due to ranking changes (Burdon, 1977). The unfavorable genetic correlations found between diameter and branch size suggests that selection for increased diameter will tend to increase branch size. This genetic correlation was similar to the estimate of 0.70 found by Dungey et al. (2013) for 6-years-old *C. lusitanica* trials in New Zealand, which may be strong enough to warrant consideration when selecting for growth traits.

The negative genetic correlations found between form traits (stem straightness, malformation score, and acceptability) and canker severity were in agreement with the estimates obtained by Gea and Low (1997) in two trials of *C. macrocarpa* in New Zealand. These genetic correlations are likely driven by the fact that canker infection causes malformation and damage, resulting in decreased scores for form traits.

In the current study, the genetic correlations between canker severity score and diameter were unfavorable, indicating that selection for higher diameter will lead to increased susceptibility to canker infection. In contrast, the correlations between canker severity and branching score was favorable, indicating that selection for less branching trees will be associated with low canker severity scores. One possible explanation for the observed genetic correlation between canker severity and branching is likely because trees with more branches would have a denser canopy more conductive to disease proliferation. The favourable genetic correlation found between stem straightness and malformation score indicates that selection for high stem straightness (moderate heritability) will indirectly improve malformation score (low heritability estimate) (Falconer and Mackay, 1996; Mrode, 2014).

The findings of this study must be viewed in light of some inherent limitations. First, the study was designed for genetic evaluation purposes based on visual symptoms or damage due to naturally occurring infections, and we are unable to discriminate between pathogen resistance or the fact that trees might be temporarily escaped from pathogen infection. The second limitation is that, using size of cankers on trees is not a reliable phenotype for assessing resistance, because the variability of size of cankers may depend on the pathogen responsible for the canker infection (i.e., *S. cardinale* and *S. cupressi*) or the variability within each pathogen population with different levels of virulence (Barnes et al., 2001), or on the different times of infections occurred, or on the size of affected organ of the tree. Third, we are not able to compare the results of genetic correlations across studies, mainly because variance components including genetic and non-genetic variances, heritability estimates and correlations between traits will change as a function of environmental conditions (i.e., temperature, humidity, and light exposure), age of trees, and different levels of disease infestation rate (de la Mata et al., 2017). For example, the previous studies on cypresses in New Zealand found no or weak positive or negative genetic correlations between growth or form traits and canker severity score (Gea and Low, 1997; Dungey et al., 2013). To overcome these limitations, the current procedure of selection for canker resistance in *C. lusitanica* breeding program in New Zealand need to be re-evaluated. Currently, selection for canker resistance genotypes relies on recurrent selection for seedlings with tolerance to canker severity in progeny test trials (Dungey et al., 2013). The development of canker resistant genotypes with durable performance requires a solid knowledge of the variability of the host reaction against pathogen and the variations in the virulence among the different pathogen strains. That requires the artificial inoculations of seedlings with isolates of both populations of fungi responsible for cypress canker (i.e., *S. cardinale* and *S. cupressi*), and monitoring their response for 3–5 years, until cankers heals completely (Danti et al., 2006, 2013b; Nocetti et al., 2015), which allow to confirm the response of plants to both species of *Seiridium* and to examine whether the genotypes that are resistant to one are resistant to the other and vice versa. The procedure should be applied under wide range of environments to confirm the stability of canker resistance under different climatic conditions. Danti et al. (2006, 2013b) described the development and performance of two canker-resistant *C. sempervirens* clones planted in two different sites in Italy. From the third year of inoculations, the resistant clones showed significant less severity of canker symptoms than that of the susceptible control clone. Furthermore, resistant clones showed complete healing of cankers and complete restoration of the trunk within 4–5 years after inoculation, whereas the cankers continued to develop on the susceptible clone. This indicates the importance of using the clonal populations in the selection for cypress canker resistance in New Zealand. However, the contribution of the non-additive genetic variance to the total genetic variation and economic assessment of the cypress market are important factors to be considered before investing in clonal breeding program for *C. lusitanica* in New Zealand. Furthermore, it is important to routinely evaluate the virulence variability of the *S. cardinale* and *S. cupressi* populations in New Zealand to direct selection for cypress clones that are resistant to more aggressive strains of the pathogen. After that, restrictions should be applied to prevent the introductions of new variants of the pathogen in the future. For example, Danti et al. (2013b) reported that the virulence variability of 21 *S. cardinale* isolates collected from cankered cypresses across the Mediterranean area has been assessed by artificial inoculations on three *C. sempervirens* clones, where none of the assessed isolates caused larger damage compared with the standard *S. cardinale* that used to develop the canker resistant clones.

Heritability estimates in the current study were low to moderate indicating that a substantial portion of the variation in growth, form and severity of cypress canker among genotypes was attributable to genetic variation and breeding values can be predicted with reasonable accuracy. Thus, the selection of genotypes that can tolerate severity of cypress canker is theoretically possible in *C. lusitanica*.

Hybridization could provide a solution to overcome cypress canker. For example, Ovens Cypress hybrid cross between *C. lusitanica* and *C. nootkatensis* has an excellent form, good canker resistance, good heartwood yield, and can perform well over a wide range of environments (i.e., sites) throughout New Zealand.

## Conclusion

In the current study, heritability estimates for diameter, and canker severity score at Welcome Bay was higher than the Matata. The moderate heritability estimate for branching size indicates that selection against large branching is possible and the breeding values can be predicted with higher accuracy so that a larger response to selection could be achieved. Unfavourable genetic correlations were found between diameter and canker severity score, indicating that fast-growing trees will be more susceptible to cypress canker. Genetic correlations between sites were very close to one, suggesting weak G × E interactions. The moderate heritability found for canker severity score showed that selecting for tree genotypes with tolerance to canker severity is possible. However, more studies are required to fully understand the complex trade-offs between tree growth and canker severity, and the unfavourable genetic correlation between branching size and diameter should be considered in the cypress breeding program.

## Data Availability Statement

The datasets presented in this study can be found in online repositories. The names of the repository/repositories and accession number(s) can be found below: https://doi.org/10.5281/zenodo.4682277 (Ismael et al., 2021).

## Author Contributions

AI analyzed the data and wrote the manuscript. HD conceived and supervised the study, made substantial contributions to the interpretation of the results, and contributed to the revision of the manuscript. GTS and KF collected the data. JK made substantial contributions to the interpretation of the results and contributed. All authors contributed to the article and approved the submitted version.

## Conflict of Interest

The authors declare that the research was conducted in the absence of any commercial or financial relationships that could be construed as a potential conflict of interest.
